# “Stepping On”: Stepping Over the Chasm from Research to Practice

**DOI:** 10.3389/fpubh.2014.00148

**Published:** 2015-04-27

**Authors:** Jane E. Mahoney

**Affiliations:** ^1^University of Wisconsin School of Medicine and Public Health, Madison, WI, USA; ^2^Wisconsin Institute for Healthy Aging, Madison, WI, USA

**Keywords:** falls prevention, older adults, community-based programs, dissemination science, implementation science

## Transfer of Stepping On: From Australia to US

Stepping On is a small-group, self-efficacy based, 7-week community workshop designed to reduce falls. It addresses four major areas: strength and balance exercises, medication review, home modification, and vision. Sessions are facilitated by a trained leader and a peer co-leader. Physical therapists teach participants to perform and advance balance and strength exercises during three sessions and a pharmacist, low vision expert, and community safety expert attend one session each. A randomized controlled trial, published in 2004, showed Stepping On participants had a 31% reduction in falls compared to controls (1).

We brought Stepping On to Wisconsin from Australia in 2006, initially training nine leaders from eight counties. Training was informal; leaders read the Australian Stepping On manual and conversed with program developer, Dr. Clemson. In the original study, occupational therapists led the workshop. However, we did not require leaders to be health care professionals. Our initial results were mixed. While leaders, host organizations, and participants loved the program and it spread quickly, evaluation of 151 participants showed no reduction in falls from 6 months before the workshop to 6 months after. Was the program not suitable for community settings in the US, or did it need more development to improve fidelity of implementation?

## Repackaging Stepping On: Defining Key Elements and Addressing Fidelity

The CDC provided 4 years of funding to develop and test a Stepping On package for US national dissemination to answer that question. Following the replicating effective programs framework (2), we began by identifying the program’s key elements. These elements are not obvious because Stepping On is a complex behavior change intervention, with many activities and objectives for each session. An international panel of experts in falls, adult learning, and exercise identified key elements through the modified Delphi technique (3). Once key elements were identified, we prepared a US version of the Stepping On Leader’s Manual, trained one new leader, and implemented the program once, monitoring each weekly session to evaluate fidelity. We observed substantial fidelity lapses. For example, the leader taught (i.e., lectured) rather than facilitated, did not leave time for Q&A, and rarely encouraged participants to share experiences. Participants did not progress their exercises.

Using root cause analysis, we identified underlying causes of the fidelity lapses and mapped solutions. First, we identified three prerequisites for being trained as a Stepping On leader: (1) background as a health professional, allied health professional, or fitness expert; (2) experience facilitating an adult self-management program; and (3) professional experience working with older adults. Second, we better defined the target population for the program. Individuals who use a standard walker for indoor ambulation may be too frail to benefit from Stepping On, and may require a more individualized approach. In addition, older adults with impaired cognition may not be able to participate fully. Third, we learned that sponsoring organizations need to clearly understand what is involved in implementing Stepping On before committing to its success. With the CDC, we developed an implementation guide (4) to help agencies understand what the program entails. Ultimately, root cause analysis changed how we select, train, and coach new leaders, identify and recruit participants, and prepare organizations to implement the program.

## Development of Training Infrastructure

Once we had refined the program for national dissemination, we needed a structure to house it. We created the Wisconsin Institute for Healthy Aging (WIHA) to foster successful dissemination of evidence-based health promotion programs in Wisconsin, and national dissemination of Stepping On. WIHA now trains Stepping On leaders and master trainers, licenses organizations to deliver the program, and provides technical assistance and updates. Master trainers observe one workshop session for each new leader they have trained, providing coaching after the session to ensure fidelity. Once a leader has successfully delivered two workshops and passed a fidelity check, he/she may become trained as a master trainer.

## Successes in Reach and Effectiveness

Stepping On has been implemented in Wisconsin and 19 other states with over 7,000 older adults participating to date. Community-based organizations value the program, and WIHA’s training and coaching results in successful adoption and high-fidelity implementation. Older adults enjoy Stepping On and recruitment is relatively easy. Invited experts, once having participated, want to continue. Since we reconfigured the implementation package based on root cause analysis, the program has been highly effective. Evaluation of 2,018 participants from 2008 to 2011 showed a significant 50% reduction in falls from 6 months before to 6 months after the program.

## Continuing Challenges and Solutions

A number of challenges hamper implementation and sustainability. For example, some organizations struggle to identify leaders and guest experts for the workshop. To overcome barriers to adoption, WIHA piloted a coaching intervention to help organizations implement Stepping On. The intervention, based on a process improvement methodology called NIATx (5), was effective. In a randomized trial, counties receiving coaching had twice the increase in number of workshops in 1 year, compared to counties on the wait list (*p* = 0.056). Currently, to help organizations with start-up, WIHA provides coaching, a pre-leader training webinar, and a wide array of materials through its website (www.wihealthyaging.org). Additionally, WIHA hosts a leader listserv, quarterly newsletter, and an annual Healthy Aging Summit where leaders learn and exchange ideas with researchers, community partners, and health care providers.

Program reach is also a challenge. Implementation is limited among African-American, Hispanic, tribal, and other minority cultures. In response to this need, we are working on an adaptation, “Pisando Fuerte,” for Spanish-speaking seniors. Such adaptations are urgently needed to extend benefits of this evidence-based program. Increased funding will help expand Stepping On’s reach. Title III-D of the Older Americans Act provides minimal funds for the aging network to implement evidence-based health promotion programs. There is no reimbursement (yet) through Medicare or Medicaid, and little investment from insurance or health maintenance organizations. While increasing participant fees would help fund program implementation, it would hinder participation by low-income older adults. We need policy changes that enable all at-risk older Americans to benefit from this effective program.

## Conclusion

We have successfully translated Stepping On from research to practice. This translation has been possible only through united efforts of researchers, policy-makers, and community agencies. Such a combination of stakeholders, dubbed the “triangle that moves the mountain” (6), creates success not only for the present but also for the future. Expanding Stepping On through continued partnerships across public health, aging, health care, and injury prevention sectors is the necessary next step to achieve the goal of population level reduction in falls and related injuries.

## Conflict of Interest Statement

Dr. Jane E. Mahoney is a Co-Author of Stepping On: building confidence and reducing falls in older adults. Leader Manual, 3rd North American Edition, Freiberg Press, Cedar Falls, IA, USA.

*This paper is included in the Research Topic, “Evidence-Based Programming for Older Adults.” This Research Topic received partial funding from multiple government and private organizations/agencies; however, the views, findings, and conclusions in these articles are those of the authors and do not necessarily represent the official position of these organizations/agencies. All papers published in the Research Topic received peer review from members of the Frontiers in Public Health (Public Health Education and Promotion section) panel of Review Editors. Because this Research Topic represents work closely associated with a nationwide evidence-based movement in the US, many of the authors and/or Review Editors may have worked together previously in some fashion. Review Editors were purposively selected based on their expertise with evaluation and/or evidence-based programming for older adults. Review Editors were independent of named authors on any given article published in this volume*.
